# Assessing the Validity of the MyJump2 App for Measuring Different Jumps in Professional Cerebral Palsy Football Players: An Experimental Study

**DOI:** 10.2196/11099

**Published:** 2019-01-30

**Authors:** Victor Coswig, Anselmo De Athayde Costa E Silva, Matheus Barbalho, Fernando Rosch De Faria, Claudio D Nogueira, Mariane Borges, Jéssica R Buratti, Ivaldo B Vieira, Francisco Javier López Román, José I Gorla

**Affiliations:** 1 Faculty of Physical Education Federal University of Pará Campus Castanhal Castanhal Brazil; 2 Faculty of Physical Education and Dance Federal University of Goiás Goiânia Brazil; 3 Faculty of Physical Education Department of Adapted Physical Education Studies University of Campinas Campinas Brazil; 4 Doctoral Program in Health Sciences Health Sciences Universidad Católica San Antonio de Murcia Múrcia Spain; 5 Director de la Cátedra de Fisiología del Ejercicio Health Sciences Universidad Católica San Antonio de Murcia Múrcia Spain

**Keywords:** cerebral palsy football, jump performance, mobile apps, mobile phone, paralympic sports

## Abstract

**Background:**

Vertical jumps can be used to assess neuromuscular status in sports performance. This is particularly important in Cerebral Palsy Football (CP Football) because players are exposed to high injury risk, but it may be complicated because the gold standard for assessing jump performance is scarce in field evaluation. Thus, field techniques, such as mobile apps, have been proposed as an alternative method for solving this problem.

**Objective:**

This study aims to evaluate the reliability of the measures of the MyJump2 app to assess vertical jump performance in professional CP Football.

**Methods:**

We assessed 40 male CP Football athletes (age 28.1 [SD 1.4] years, weight 72.5 [SD 6.2] kg, and height 176 [SD 4.2] cm) through the countermovement jump (CMJ) and squat jump (SJ) using a contact mat. At the same time, we assessed the athletes using the MyJump2 app.

**Results:**

There were no significant differences between the instruments in SJ height (*P*=.12) and flight time (*P*=.15). Additionally, there were no significant differences between the instruments for CMJ in jump height (*P*=.16) and flight time (*P*=.13). In addition, it was observed that there were significant and strong intraclass correlations in all SJ variables varying from 0.86 to 0.89 (both *P*<.001), which was classified as “almost perfect.” Similar results were observed in all variables from the CMJ, varying from 0.92 to 0.96 (both *P* ≤.001).

**Conclusions:**

We conclude that the MyJump2 app presents high validity and reliability for measuring jump height and flight time of the SJ and CMJ in CP Football athletes.

## Introduction

Cerebral Palsy Football (CP Football) is a paralympic sport played exclusively by athletes with central neurological injuries, such as cerebral palsy (CP), traumatic brain injuries, or stroke [1]. CP Football players may have ataxia, hypertonia, or dystonia and are divided into classes based on their functional profile levels [1]. The concepts of the sport are quite similar to mainstream football, except for the use of 7 players, smaller field measurements, shorter duration of matches (30 minutes per time), and the lack of the offside rule [1]. Due the intermittent characteristic of the sport, and the fact that moments of high intensity are decisive in the game [2], CP Football has similar strength and power demands to mainstream soccer [3,4].

In addition, CP Football shows a relatively high injury rate. For example, injury rates for CP Football in the Rio 2016 Paralympic Games were 12.7/1000 athletes/day [5], in which it was noted that injuries by overuse had the second highest prevalence (4.5%) and higher injury rate during competition (34.5%). This is quite similar to the findings by Derman et al [6] who presented larger incidences of injury rates in CP Football (18.8/1000 athlete days) in comparison to all sports (12.1/1000 athlete days) in the London 2012 Paralympic Games.

There are many factors related to injury rates and sports performance in CP Football. From these, strength and power have been highlighted as relevant parameters for injury prevention [7,8] and training monitoring [9]. In this sense, it is important to establish reliable options for measuring and analyzing these variables, since vertical jumps are considered an ecological alternative provided in field assessments of lower-limb strength and power. Furthermore, increasing vertical jump assessment options could encourage and help coaches and trainers monitor and prescribe training based on this objective external load parameter, which has already been demonstrated to be a relevant factor in CP Football [5].

To accurately evaluate vertical jumps, expensive instruments with low portability are often needed, such as force platforms [10] and contact mats [11-12]. In this way, the demand for evaluation methods using mobile devices has increased. In addition to the technological advancement of smartphones, mobile apps have rapidly evolved from a trend [13] to a well-stablished part of sports and exercise medicine in constant and fast evolution [14]. Therefore, the MyJump2 app has become a viable option for evaluating jump heights. In addition, this app promises to provide quick and immediate data acquisition, allowing for easy monitoring in virtually any environment [15], and has already shown high reliability and reproducibility in vertical jumps when compared to force platforms and high-speed video cameras [16-18]. However, due to the specificities of CP athletes, such as asymmetries or involuntary spasms [6], it became necessary to test the validity and reliability of this mobile app in CP populations and sports. In this context, this study aimed to evaluate the reliability of the measures of the MyJump2 app to evaluate vertical jump performance in professional CP soccer athletes.

## Methods

### Experimental Design

To evaluate the reliability of the app, we recruited professional CP Football athletes during the 2017 Brazilian CP Football Championship. For this, the board of the National Association of Sport for the Disabled (NASD, Brazil) authorized the study. The directors of each team were clarified about the research proposal and methods and allowed the researchers to perform data collection with their athletes. First, we informed all subjects about the risks, benefits, and discomforts of participation by signing the consent form, and data collection occurred in one session. The study followed the ethical principles stated in the Declaration of Helsinki and was approved by the local ethics committee (#2.475.044). The athletes performed the countermovement jump (CMJ) and squat jump (SJ) and were assessed by 2 instruments simultaneously (a contact mat and MyJump2). Each participant performed 3 repetitions of each jump, and the highest height values were used in the analyses. The CMJ and SJ tests were performed in a circuit arrangement to guarantee similar rest time between the efforts.

Before data collection, subjects performed a specific warm-up for 5 minutes, which involved the execution of vertical jumps similar to those applied in the evaluations, in order to learn the how the jump would be executed and with stimulation of slow and fast cycles of stretching and shortening. After the specific warm-up period, participants were instructed to perform 3 CMJs with their hands fixed at the waist, performing the jump at the highest possible height [9]. All participants received verbal guidance to make the highest jump possible. There was no additional verbal stimulus to avoid differences between subjects.

### Sample

A sample size calculation from an earlier investigation indicated that 7 participants would be needed, considering *P*=.001 and a power of 90% [16]. Thus, for this study, 40 male athletes (28.1 [SD 1.4] years, 72.5 [SD 6.2] kg, and 176 [SD 4.2] cm) without presenting acute or chronic conditions that prevented them from performing the jump protocol were included. From these, 30 were hemiplegic, 9 were diplegic, and 1 was monoplegic. Inclusion criteria required that participants have neurologic injuries at the central nervous system, be engaged in official professional CP Football competitions, and have prior experience in the vertical jumping exercise, which means that they performed plyometric exercises during training and were engaged in frequent jump tests during periodic evaluations. We excluded participants who did not complete the jumps for any reason, presented pain or injury, did vigorous physical activity, or had ingested central nervous system stimulants (ie, caffeine beverages) during the data collection phase (n=0).

### Procedures

#### Countermovement Jump

In the CMJ, the individual starts in an orthostatic position with the hands fixed at the waist and, at the evaluators' command, performs a squat until the knees reach an angle of 90°. Then, the participant extends the hips and the knees to project the body vertically with the greatest speed and strength possible to reach the maximum possible height [19]. Participants were instructed to not flex the knee or dorsiflex the ankle during the flight phase. All participants received verbal stimuli for a better performance. A 30-second rest interval between each jump was set.

#### Squat Jump

In the SJ, the individual starts in an orthostatic position and, at the evaluator´s command, performs a squat until the knees reach a 90° angle. This position is maintained in isometric contraction for 3 seconds, after which the individual extends the hips and knees to project the body and the load vertically at the highest possible speed and strength, that is, to achieve maximum power during the execution [19].

#### Contact Mat

The Jump System Pro Contact Mat (Cefise) evaluates the power output through flight time. Output data were collected by the Jump System Pro Software, version 1.0 (Cefise). The contact map showed high reliability for jump height, with an intraclass correlation coefficient (ICC) of 0.91 and a coefficient of variation of 10% [20].

#### MyJump2 Application

The app for the iOS operating system (Apple Inc) [17] was developed using the XCode0.5 software for Mac (OSX 10.9.2, Apple Inc) and installed on the iPhone 6s (Apple Inc). The evaluation required a high-speed camera (120 Hz) with a minimum resolution of 720p. The app analyzed the height of the vertical jumps by calculating the time between 2 frames (in ms) selected by the evaluator, corresponding to the loss and return of contact to the ground. For this instrument, the same evaluator performed all the collections and was always in the same position (at the front) and at the same distance (1.5 m) from the material being evaluated. For interevaluator and intraevaluator reliability, recorded videos were analyzed by 2 evaluators (inter) and one of them repeated the procedure after 1 week (intra).

### Statistical Analysis

Data are presented in mean (SD). The Shapiro-Wilk test was used to assess normality, and the 2-tailed paired *t* test was used to compare instruments. For the reproducibility of the test measurements, the ICC, SE of measurement, that is, SEM=SD × √(1−ICC), and minimal detectable change, that is, MDC=SEM × 1.96 × (√2), were applied [21]. The Pearson correlation coefficient was applied for the correlations, and Bland-Altman plots were applied to test the level of agreement between instruments. All analyses were performed using SPSS software (IBM SPSS Statistics, version 20.0). For all variables, statistical significance was set at *P* ≤.05.

## Results

The mean and SD of jump height and flight time of squat jump and countermovement jump were assessed using MyJump2 and a contact mat in 40 male CP Football athletes. Table 1 shows the values of the absolute comparison and ICC between the instruments for the jump height and flight time of the SJ and CMJ. There were no significant differences between the instruments in the jump height (*P*=.12) and flight time (*P*=.15) variables. The effect size of the 2 variables was trivial for jump height and flight time, according to the Cohen classification [22]. There were no significant differences between the instruments for the CMJ in the jump height (*P*=.16) and flight time (*P*=.13) variables. The effect size of the 2 variables was also trivial for jump height. In addition, it was observed that there were significant intraclass correlations in all SJ variables (*P*<.001). Strong correlations were found between jump height and flight time (ICC=0.89 and 0.86, respectively), being classified as “almost perfect” [22]. Similar significant intraclass correlations were observed in all variables from the CMJ (*P* ≤.05), where jump height and flight time presented excellent levels varying from 0.92 to 0.96, classified as “near perfect” [22].

**Figure 1:**
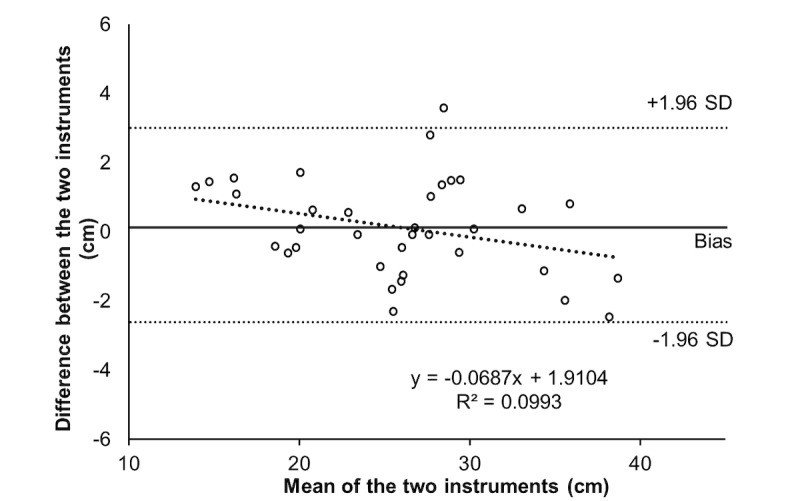
Bland-Altman for agreement analysis of squat jumps.

**Figure 2:**
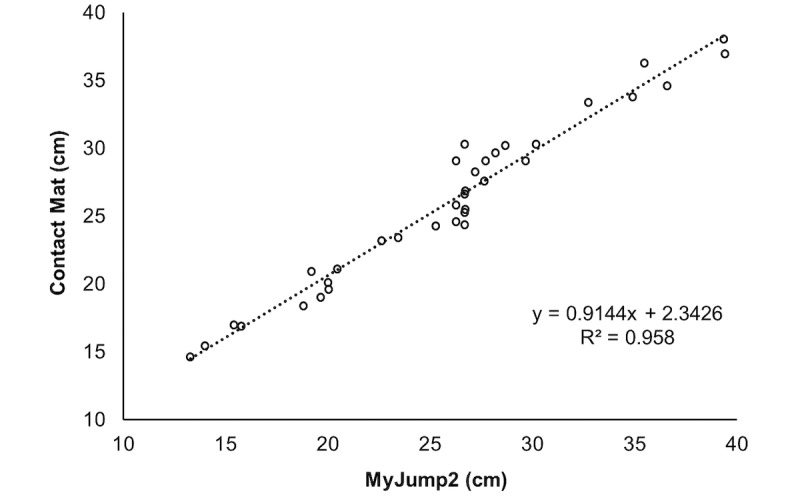
Correlation for agreement analysis of squat jumps.

**Figure 3:**
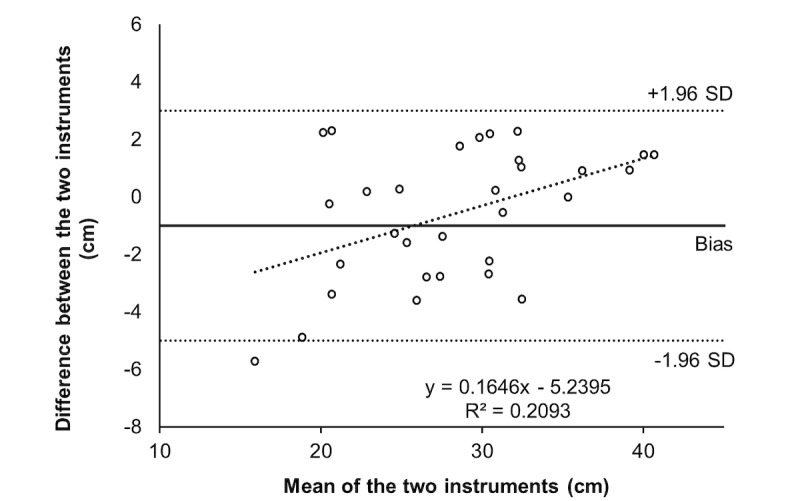
Bland-Altman for agreement analysis of countermovement jumps.

**Figure 4:**
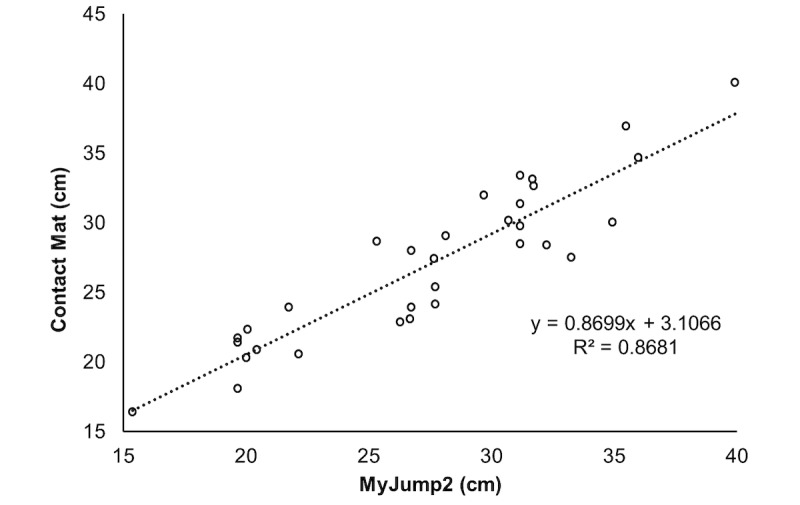
Correlation for agreement analysis of countermovement jumps.

Table 2 presents reliability data between evaluators, while Table 3 presents values between test and retest. For both, high values of intraclass correlation and very low values of standard error of measurements and minimal detectable changes were found. Bland-Altman and correlation analysis are presented in Figures 1 and 2 for SJ and in Figures 3 and 4 for CMJ, respectively. For both jumps, high levels of agreement were found, and the differences were similar for all ranges of heights.

**Table 1:** Values of absolute comparison and intraclass correlation coefficient between the instruments for the jump height and flight time of squat jump and countermovement jump.

Jump type		MyJump2, mean (SD)	Contact mat, mean (SD)	*t (df)*	*P* value	Cohen *d* effect size	ICC^a^	*P*_ICC_^b^
**Squat jump**								
	Jump height (cm)	25.1 (7.4)	26.2 (6.2)	−1.59 (35)	.12	0.17	0.89	<.001
	Flight time (ms)	448.5 (70.0)	458.67 (55.6)	−1.47 (35)	.15	0.17	0.86	<.001
**Countermovement jump**								
	Jump height (cm)	28.4 (6.5)	27.8 (6.1)	1.42 (33)	.16	0.09	0.92	<.001
	Flight time (ms)	477.7 (56.1)	473.2 (52.5)	1.27 (33)	.21	0.02	0.96	<.001

^a^ICC: intraclass correlation coefficient.

^b^*P*_ICC_: Level of significance of intraclass correlation coefficient, which was set at *P* ≤.05.

**Table 2:** Interevaluator intraclass correlation coefficient of squat jump and countermovement jump measurements in the MyJump2TM App.

Jump type		Evaluator 1, mean (SD)	Evaluator 2, mean (SD)	ICC^a^	*P* value^b^	Standard error of measurement	Minimal detectable change (%)
**Squat jump**							
	Jump height (cm)	25.5 (7.0)	23.3 (7.1)	0.93	<.001	0.56	0.79 (3.09)
	Flight time (ms)	452.5 (66.0)	442.52 (83.5)	0.90	<.001	7.45	10.53 (2.33)
**Countermovement jump**							
	Jump height (cm)	28.4 (6.7)	27.1 (7.8)	0.95	<.001	0.36	0.51 (1.81)
	Flight time (ms)	477.7 (57.5)	468.7 (71.3)	0.92	<.001	5.12	7.24 (1.51)

^a^ICC: intraclass correlation coefficient.

^b^Level of significance set at *P* ≤.05.

**Table 3:** Intraevaluator intraclass correlation coefficient of the MyJump2 in squat jump and countermovement jump.

Jump type		1^st^ analysis	2^nd^ analysis	ICC^a^a	*P* value^b^	Standard error of measurement	Minimal detectable change (%)
**Squat jump**							
	Jump Height (cm)	25.5 (7.0)	24.8 (7.4)	0.99	<.001	0.07	0.10 (0.39)
	Flight Time (ms)	452.48 (66.02)	447.8 (70.2)	0.95	<.001	4.08	5.77 (1.27)
**Countermovement jump**							
	Jump Height (cm)	28.4 (6.7)	28.1 (6.6)	0.99	<.001	0.04	0.06 (0.20)
	Flight Time (ms)	477.7 (57.5)	477.4 (56.1)	0.99	<.001	0.06	0.08 (0.01)

^a^ICC: intraclass correlation coefficient.

^b^Level of significance set at *P* ≤.05.

## Discussion

### Principal Findings

The main objective of this study was to assess the reliability of a mobile app that measures jumping performance in CP Football athletes to establish a reliable field evaluation possibility for jump analysis. In this sense, we assessed 40 male, physically disabled players using the MyJump2 app and a contact mat simultaneously. Additionally, 2 evaluators made the measurements with the app, and one of them repeated the measurement 1 week later to analyze the interevaluator and intraevaluator variability. Our main results showed that MyJump2 is a reliable and valid method compared with the contact mat when assessing jump height and flight time.

### Comparison With Prior Work

MyJump2 seems to be reliable for assessing jump height and flying time in CP Football. Our results are in agreement with 2 other studies that investigated MyJump reproducibility in vertical jumps [16,17]. Balsalobre-Fernandez et al [17] evaluated the app’s validity, compared with a force platform, to assess CMJ in 20 recreationally active healthy men and observed a near perfect correlation in jump height. Thus, the authors indicated that CMJ could be easily measured and was reliable and reproducible through the app.

The other study on the theme [16] had analyzed different jumps (drop jump, SJ, and CMJ) in a sample of 21 male and female athletes, and the authors compared the app with a contact platform and a high-speed video camera method. In all jumps, there was a strong and significant correlation between the instruments. Similarly, our results showed that the CMJ has a strong correlation with the reference method in jump height (*r*=0.95).

Other relevant results are about the interevaluator and intraevaluator reliability. Our results show that the MyJump2 is reproducible when used by different subjects and at different occasions. These results are in agreement with literature about MyJump2 reproducibility [17].

Additionally, in competitive periods when it is not possible to use the gold standard equipment, the app seems to be a good alternative for evaluating athletes with neurologic damage who would benefit from frequent monitoring for training loads and adaptations [23-24] and also for soft-tissue lesion risk [25,26]. Given the portability and practicality of MyJump2, smartphones can quickly become a standard method for assessing physical performance in the field with great precision in CP Football.

The comparison between MyJump2 and other methods, such as force platforms [14-16] and the field method (Vertec) [27], is important to consolidate the app. This is justified by the use of a few force platforms in field evaluations, which raises the importance of the comparison between MyJump2 and other field methods [27]. In this regard, our study compares the app with one of the most used field techniques, the contact mat. Compared with force platforms, contact mats can be used in a wide variety of scenarios, and this can be considered a more ecologic option in agreement with the study that analyzed a comparison between MyJump2 and Vertec [27]. Another factor that deserves further comment is the type of jumps assessed here. The CMJ and SJ are consistent with the current literature [9,16,18,28,29].

Another important aspect of this study is that it appears to be the first work to use the app in a paralympic sport. In general, paralympic sports has a particular aspect beyond those related to training: the functional classification. To reduce subjectivity, it is necessary that the functional classification be evidence based [30]. In this sense, physical assessments are lacking and should be increased, including that for strength and power [31]. Thus, the implementation of mobile apps seems to be interesting in the evidence-based classification of paralympic sports. Another interesting characteristic of this paper concerns the sample. In the paralympic context, it is particularly challenging to do investigations with a large number of subjects, especially at higher competitive levels. Therefore, this study used a significant paralympic sample from a top national ranking team, which reinforces the originality and relevance of our findings.

### Limitations

The study has some potential limitations. Due to the fact that data collection was conducted during a Brazilian CP Football Championship, it was not possible to use a better reference method, such as the force platform. Despite that, it could be considered a methodological choice in order to raise the ecological validity of our findings. With our study design, it was not possible to assess the use of the app in other conditions, such as before and after the games, to analyze the applicability of the app, which we suggest for further studies.

### Future Directions

Since the MyJump2 app is a reliable method for assessing jump height in CP Football, future investigations may be designed in some topics differing from validation studies. With the limitations of laboratory methods, there is a trend toward using small samples in investigations, and less field assessments are observed. Therefore, one possibility will be to assess a wide sample to establish reference values of jump height and flight time in CP Football, using as large a sample as possible. Other possibilities may be to use prospective assessments of jump performance and its association with injury prevention parameters in this population, as well as assessments of performance levels and pregame, postgame, and competition recoveries. Regarding validation studies, it seems to be an important possibility for the validation of MyJump2 to assess other types of jumps besides CMJ and SJ. For example, the asymmetry jump test, drop jump test, and horizontal jump are three kinds of skills that can be used to assess jump performance and, to this date, have not been validated in CP Football evaluation.

### Conclusions

Thus, we conclude that the MyJump2 app presents high validity and reliability to measure the jump height and flight time of the SJ and CMJ in elite CP Football athletes. Our findings suggest that this tool can be very useful in jump performance analysis of the Paralympics. In addition, we believe that our findings could encourage trainers, coaches, and athletes to monitor jump performance, which is relevant information to improve decision making in training control and prescription.

## Abbreviations

CMJ: countermovement jump
CP Football: Cerebral Palsy Football
CP: cerebral palsy
ICC: intraclass correlation coefficient
MDC: minimal detectable change
SEM: standard error of measurement
SJ: squat jump
